# The Public Health Services

**Published:** 1919-09-06

**Authors:** 


### THE PUBLIC HEALTH SERVICES.

The time of trial, as also the time of opportunity, for the public medical services of this country has come. That work of after-war "reconstruction," of which so much has been heard, has hastened an epoch in the evolution of public health organisation and a Ministry of Health is in being.

The demand for a Ministry of Health has been rightly based upon the pressing need for unification and co-ordination, procedures difficult at all times and in all affairs, and supremely difficult in the case of the work which the new Ministry will be called upon to do. The Act under which it has been created has provided but a framework: it has, as it were, surrounded various discrepant, jealously separated, rambling and inconvenient departments, which the piecemeal legislation of nearly fifty years has thrown together and never completed, with a new scaffolding, lightly and hurriedly constructed, within which a beautiful and harmonious new building is to be produced, largely from the old materials. The simile, however, is incomplete. The importance and the difficulties of the work which lies before the Ministry are immense. The real significance of the creation of this new body in the State is that it is a Ministry of Health and not merely a Ministry of *Public* Health; it will concern itself not only, perhaps ultimately not chiefly, with preventive medicine, but with treatment, whether institutional or domestic, with eugenics, with the welfare of all classes of the community. Its influence will rapidly extend and will be felt in connection with medical and nursing education, with our great system of voluntarily-maintained hospitals, and with the work of the medical practitioner. Its responsibilities are only equalled by its opportunities.

The anomalous conditions which have arisen with regard to institutional medical and surgical treatment are well known. Until about fifty years ago our Governments left the question of the health of the people somewhat severely alone (such matters as vaccination excepted), and the only provision for medical treatment was that made as the result of charity and that scamped and discredited Poor-Law Service, which was so scathingly satirised in the pages of the Victorian novelists. Three-quarters of the nineteenth century, that wonderful century of progress and prosperity, had elapsed before the public health was definitely made the affair of the State. Isolation hospitals for the treatment of infectious diseases were opened broadcast, but the treatment afforded (as was already the case in dealing with the insane) was only incidental, following inevitably upon hospital segregation. Meanwhile, with advances in medical and surgical treatment and in nursing, the demand for in-patient hospital treatment everywhere, and especially in the great towns, vastly outgrew the hospital accommodation; surgical anæsthesia and asepsis revolutionised hospital treatment—no adequate preparation was made to meet the change. There was, however, one department of public service, and one only, which had available the means and could evolve the machinery to establish something resembling a State hospital system: that Cinderella of our social administration, the Poor-Law system, in the process of improving its treatment of the sick poor, has (despite itself) carried that improvement so far that it has come to possess the only State hospitals in the kingdom, hospitals in most instances already fairly well staffed and excellently equipped. This is the ever-puzzling anomaly, we might say absurdity, which the Ministry of Health is called upon to remove once and for all: that the great proportion of the beds available for the treatment of the sick in this country are under the control, not of any properly constituted hospital authority, but of Guardians of the Poor, who were not appointed to provide, are not fitted to control, and ought never to have come to be associated with State hospitals. Something of the degrading influence of pauperism will attach to these institutions until they are removed from Poor-Law control and associations. The ground has been fully prepared for the change by the Reports of the Royal Commission in 1909 and the Ministry of Reconstruction Report on the Transfer of Functions of Poor-Law Authorities in England and Wales in 1918. The first action of the Ministry, and it is one most urgently called for, should be to bring about that reform of the Poor Law which has been so much talked of and is so long overdue.

The theoretical restriction of State departments to the preventive side of medicine has now passed. The medical examination of school children provided the thin end of the wedge, and it needed no undue optimism to foresee that provision of treatment would necessarily follow—as it has now done. In the steps which have been taken with a view to the control of tuberculosis and of venereal disease prevention and treatment go, perforce, hand in hand. This is also the case with regard to maternity and infant welfare work, the developments of which form the latest and most active interest of public health work. The medical and nursing professions are vitally interested in the further evolution of the Ministry of Health.

The influence of the new department in the matter of medical appointments will ultimately be great and must tend towards co-ordination. How nearly, or how speedily, this co-ordination will approach the establishment of a State Medical Service it is impossible for us to discuss, much less to attempt to foretell. At present the only appointments which fall strictly within the sphere of the public health services are those directly under the Ministry and the County and District Medical Officerships of Health, with the Tuberculosis and School Medical Officerships. The appointments so far made to the direct service of the Ministry have been largely filled by the transfer of those holding office under the old departments: it cannot be said that the selection has erred on the side of preferring medical men, a considerable proportion of those appointed being women! There is no immediate likelihood of any great or rapid increase in the medical staff of the Ministry. For the Medical Officerships of Health the outstanding qualifications are still the possession of sound medical training and experience and of a diploma in public health or its equivalent: we once again commend to students and young practitioners the acquisition of such a diploma, the course of study for which is in itself a most valuable and interesting, to some even a fascinating, educational exercise. The appointment of school medical officer has improved in status and remuneration, is by no means devoid of interest, and affords useful experience, with, often, an insight and introduction to the work of a medical officer of health. The tuberculosis service is recruited from men of general, as well as special, experience: somewhat starved during the war, the service is now again actively engaged and its special attention is being devoted to providing for discharged tuberculous service men and women.

The report of the Inter-departmental Committee on Tuberculosis (Sanatoria for Soldiers), which has recently been published, is of interest in this connection. The report shows that fears of a wide dissemination of tuberculosis as a result of war-conditions have, fortunately, not been realised; some 35,000 "ex-service men" are suffering from tuberculosis, of whom a proportion would have become infected under civilian conditions of life, whilst many had been previously tuberculous and are suffering from re-infection or relapse. Another subsidiary medical service is that of the fever hospitals, which hospitals, again anomalously, are in London in charge of the Metropolitan Asylums Board, itself an offshoot from the Poor-Law service. These appointments, like those in the Poor-Law hospital or infirmary, afford to young practitioners experience of high value and of a character not obtainable elsewhere. We have already said that the Poor-Law medical service is, or soon should be, in a state of transition: the work is done under good conditions, and, whereas a generation ago junior posts were accepted for the value of the experience at a nominal salary, or even without salary, good salaries are now offered. In these institutions much side-track work has (*inter alia*) been done, which must later develop and interlock with the work of the school clinic and the maternity and infant welfare centre, and which is likely to prove in the future of enhanced value as practical experience. In fact the prospects offered by these appointments have improved and are improving, and this statement applies also to the hitherto somewhat unpopular junior asylum posts: nor must the probability of better opportunities being offered in future to bacteriological and pathological laboratory workers be forgotten. Maternity and infant welfare work will ultimately engage increasing numbers of whole-time medical officers, and such posts will be, of course, frequently filled by medical women. It is unlikely that the Ministry of Pensions will for long require the services of any considerable number of medical officers for whole-time work; the maintenance of a special service by this Ministry would involve once again a useless and wasteful overlapping, and the work entailed will be principally such as can best be carried out by referees selected on the ground of specialist experience.

The future official relationships of the District Medical Officer, the panel-doctor, and the general practitioner rest on the knees of the gods: whether, in future years, we shall be called upon to include some or all of their present activities as falling within the scope of the public medical services under the Ministry of Health time alone will show.

